# Supplementary material for: Late Neurological Consequences of Zika Virus Infection: Risk Factors and Pharmaceutical Approaches
Source: Pharmaceuticals (Basel). 2019 Apr 17;12(2):60. doi: 10.3390/ph12020060 (PMC6631207; doi:10.3390/ph12020060)
Supplement: Supplementary file 1 [file pharmaceuticals-12-00060-s001.pdf]

Supplementary

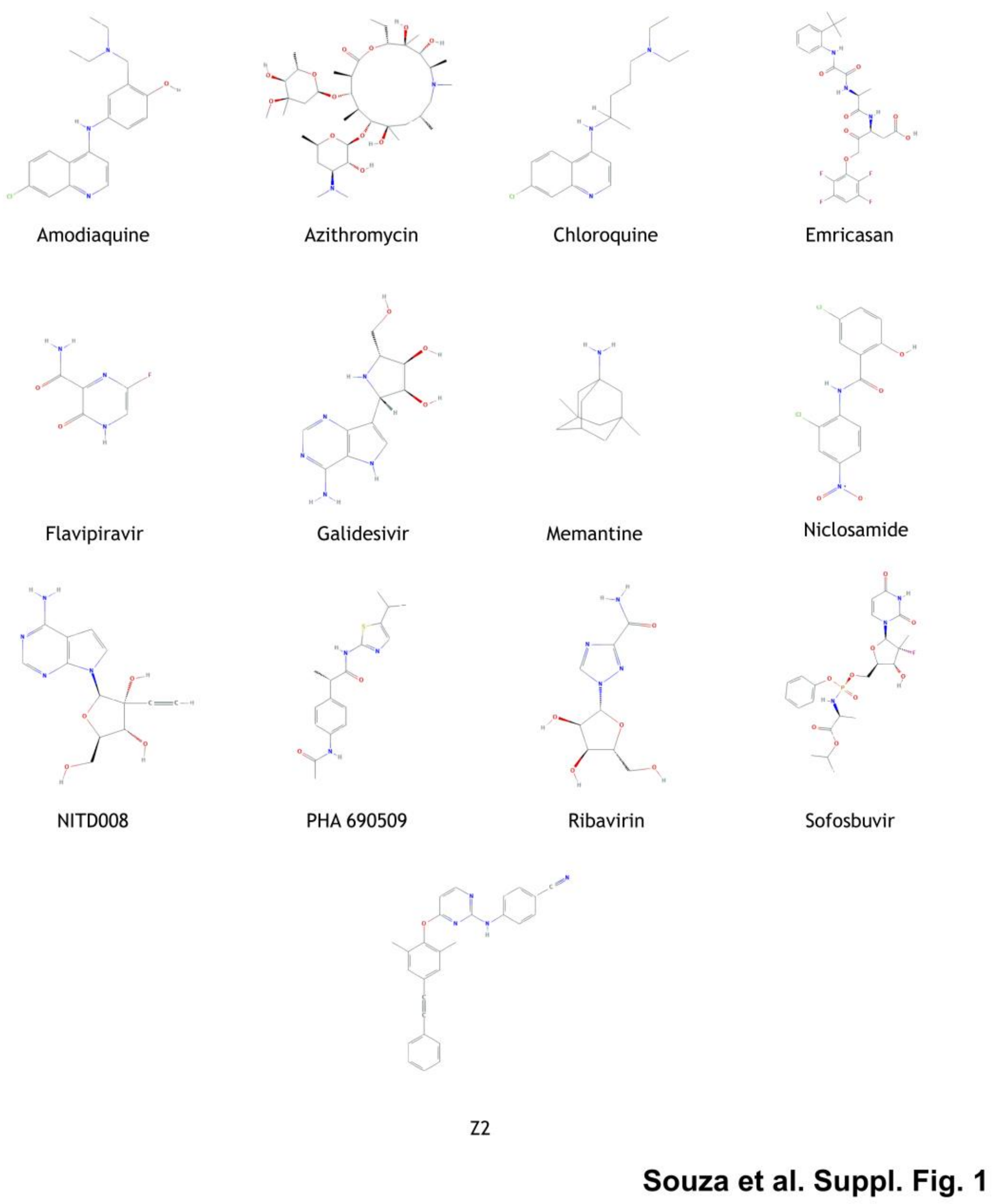

Figure S1. Possible candidates for ZIKV infection treatment. These drugs, either repurposed or under development, have shown promising results in treating ZIKV infection in both adult and vertical transmission or neonatal models (see text). All structures from Pubchem Compound Database [139].
